# Cancer Immunology and Immunotherapy

**DOI:** 10.1155/2015/393454

**Published:** 2015-06-24

**Authors:** Mohammad Owais, Swaleha Zubair, Anshu Agrawal, Yung-Fu Chang

**Affiliations:** ^1^Aligarh Muslim University, Aligarh, India; ^2^University of California, Irvine, USA; ^3^Cornell University, Ithaca, USA

The study of molecular and cellular interplays between immune system and cancerous cells is gaining tremendous momentum across the globe. Concomitantly, with the better insight into the intricacies of cancer immunology, immunotherapeutic approaches to deal with cancer have garnered tremendous boost in the recent past; reckoning with these, it is timely to analyse their potentialities either as standalone stratagem or in conjunction with traditional cancer therapeutic modalities. The comprehensive review by M. L. Santangelo et al. entitled “Immunosuppression and Multiple Primary Malignancies in Kidney-Transplanted Patients: A Single-Institute Study” provides an overview of immunotherapeutic interventions for metastatic renal cell carcinoma (RCC) as well as updating the readers on the recent developments in the field.

Further, the article entitled “Immunotherapy for Bone and Soft Tissue Sarcomas” by T. Uehara et al. enlightens the readers on immunotherapeutic strategies against bone and soft tissue sarcomas and metastatic prostate cancer under various stages of trials, besides highlighting their roles as an adjunct to traditional therapeutic modalities.

Natural killer (NK) cells have long been hypothesized to play a pivotal role in the development of new immunotherapeutic strategies to combat variety of cancers. In this regard, the article titled “‘Adherent' versus Other Isolation Strategies for Expanding Purified, Potent, and Activated Human NK Cells for Cancer Immunotherapy” by S. R. Selvan and J. P. Dowling introduces a simple methodology for isolation and expansion of NK cells for adoptive cell therapies. Moreover, the researchers also equate potentialities of the newly introduced method with various published protocols to underline its effectiveness thereof. In the arena of NK cell based immunotherapies, the article “NKG2D and DNAM-1 Ligands: Molecular Targets for NK Cell-Mediated Immunotherapeutic Intervention in Multiple Myeloma” by C. Fionda et al. has come up with satisfactory results to further potentiate NK cell based immunotherapies. In their article of this special issue, the authors collate and discuss the molecular pathways whereby various chemotherapeutic agents could regulate the expression of NK cell activating ligands in multiple myeloma cells. Further, in a manner similar to NK cells, dendritic cells are also exploited in adoptive cell therapies; the manuscript “Dendritic Cell-Based Immunotherapy Treatment for Glioblastoma Multiforme” by L. Yang et al. discusses DCs based immunotherapeutic interventions for glioblastoma multiforme.

It is in general consensus that immunodeficiencies are associated with higher risk of cancer susceptibility; nevertheless, there remains paucity of reports on the association of immunodeficiencies with the development of multiple primary malignancies. In this regard, the study by R. Raman and D. Vaena illuminated the relationship between immunodeficiency status of the patient, related to kidney transplant in particular, and occurrence of multiple primary malignancies; nonetheless, further evidences are required to firmly establish the linkages between immune status of the recipient and its correlation with incidences of malignancy.

With continuous efforts laid down to better dissect the interplay between immune system and tumors, significant progresses have been made in the recent past, albeit much have been unveiled; nevertheless, the drive continues to explore more and more. To this end, the manuscript by N. Vigneron provides a better insight into the ins and outs of tumor-immune system interrelationships highlighting the recent understandings gained in the field. Further, the article by S. Stigliani et al. suggests that expressions of FOXP3, CD14, and ARG1 in neuroblastoma tumor tissue from high-risk patients are significantly associated with event-free and overall survival. Besides, C. Li et al. investigate the association of CXCL13 (C-X-C motif chemokine 13) with hepatocellular carcinoma (HCC) and the authors further suggest that the correlation of CXCL13 with progression of HCC is related to the activation of Wnt/*β*-catenin pathway and the facilitation of IL-12, IL-17, and IgG4. Ascertaining their role in progression of HCC, the authors anticipate that CXCL13 could be a potential target for the diagnosis and treatment of HCC. Further, the report by A. Curioni-Fontecedro et al. highlights the intratumoral heterogeneity of MAGE-C1/CT7 and MAGE-C2/CT10 expression in mucosal melanoma. The article by Y. Nishimura et al. investigates the immunological effects of asbestos exposure and analyzes immune functions of patients with mesothelioma, thereby signifying that there occurs functional alteration in natural killer cells and cytotoxic T lymphocytes upon asbestos exposure as well as in malignant mesothelioma patients, while the manuscript by Z. Liu et al. investigates the regulative effects of microRNA-451a (miR-451a) on cell proliferation and sensitivity to tamoxifen in breast cancer cells. Further, P. Johnson et. al. from Cancer Research UK Clinical Centre have highlighted that it is equally important to delineate the metrics that are appropriate to annotate the significance of new cancer therapeutics modalities. The study also provided an insight into the intricacies that are the same and further ascertains that the mean overall survival, cure fraction, and overall survival rate at landmark time points represent the more appropriate endpoints.

The recent conceptual and technical footing of cancer immunology has paved ways to discover innovative cancer immunotherapies to treat and retard progression of the disease. It is widely accepted that the gamut of genetics and epigenetics changes occurring in tumors provides diverse set of antigenic repertoire that the immune system can exploit to distinguish tumor cells from their normal healthy counterparts. Moreover, studies continue to explore various genetic factors that increase the risk for cancer; the article by Y. Liu et al. studied the polymorphisms of nuclear factor-kappa B (NF*κ*B) and its inhibitor (I*κ*B*α*) and their synergistic outcome on nasopharyngeal carcinoma (NPC) predisposition. From their study, authors anticipate that genetic variants in NF*κ*B1 (rs28362491del>ins ATTG) and I*κ*B*α* (rs696G>A) and their synergistic outcome contribute to NPC susceptibility. Further, the manuscript by I. Silvestri et al. embarks on the importance of insight into the intricacies of antigenic peptide presentation in immunotherapy as well as in vaccine delivery.

Reckoning with the recent efforts devoted to developing superior strategies to fight against various diseases, over the years, there has been great wave of enthusiasm regarding employment of immunomodulators to combat various untamed diseases. In fact the strategy is high on pharma agenda and various immunomodulators especially naturally derived agents have been explored against various ailments including cancer. Considering the impact of immunomodulators in the field on cancer immunotherapies, W.-J. Wang et al. illuminated the role of mushroom *β*-Glucan to immunomodulate tumor associated macrophages in Lewis Lung Carcinoma. Moreover, the article by A. Ito et al. updates the readers on the clinical development of immunomodulators or immune checkpoint inhibitors. Further, the manuscript by Q. Guo et al. delineates the effect and molecular mechanisms of traditional Chinese medicine (TCM) on regulating tumor immunosuppressive microenvironment (TIM) revealing bidirectional and multitargeting features of TCM on TIM.

Finally, the pioneer work by O. Kurtenkov et al. for the first time revealed the fact that increased sialylation of anti-Thomsen-Friedenreich (TF) Antigen (CD176) antibodies is strongly linked to gastric cancer; reckoning with this, the authors anticipate that the important biomolecule can be employed as a novel biomarker for cancer detection and prognosis.

Besides active regimens to control and cure one of the most dreadful diseases across the world, the immunological intervention to dissipate various forms of the cancer seems to be the promising ordeal. The timely coverage of the various cancer cells versus host immune system interplay by some of the leading experts of the field will certainly take us to the state of affairs that will offer a great input to control this important ailment.

*Mohammad Owais* *Swaleha Zubair* *Anshu Agrawal* *Yung-Fu Chang*

